# Thermal Expansion and Rattling Behavior of Gd-Filled Co_4_Sb_12_ Skutterudite Determined by High-Resolution Synchrotron X-ray Diffraction

**DOI:** 10.3390/ma16010370

**Published:** 2022-12-30

**Authors:** João E. F. S. Rodrigues, Javier Gainza, Federico Serrano-Sánchez, Romualdo S. Silva, Catherine Dejoie, Norbert M. Nemes, Oscar J. Dura, José L. Martínez, José Antonio Alonso

**Affiliations:** 1 Instituto de Ciencia de Materiales de Madrid (ICMM), Consejo Superior de Investigaciones Científicas, Sor Juana Inés de la Cruz 3, E-28049 Madrid, Spain; 2 European Synchrotron Radiation Facility (ESRF), 71 Avenue des Martyrs, 38000 Grenoble, France; 3 Department of Physics, Federal University of Sergipe, São Cristóvão 49100-000, SE, Brazil; 4 Departamento de Física de Materiales, Universidad Complutense de Madrid, E-28040 Madrid, Spain; 5 Departamento de Física Aplicada, Universidad de Castilla-La Mancha, E-13071 Ciudad Real, Spain

**Keywords:** skutterudites, CoSb_3_, thermal expansion, rattling effect, thermoelectrics

## Abstract

In this work, Gd-filled skutterudite Gd_x_Co_4_Sb_12_ was prepared using one step method under high pressure in a piston-cylinder-based press at 3.5 GPa and moderate temperature of 800 °C. A detailed structural characterization was performed using synchrotron X-ray diffraction (SXRD), revealing a filling fraction of x = 0.033(2) and an average <Gd–Sb> bond length of 3.3499(3) Å. The lattice thermal expansion accessed via temperature-dependent SXRD led to a precise determination of a Debye temperature of 322(3) K, from the fitting of the unit-cell volume expansion using the second order Grüneisen approximation. This parameter, when evaluated through the mean square displacements of Co and Sb, displayed a value of 265(2) K, meaning that the application of the harmonic Debye theory underestimates the Debye temperature in skutterudites. Regarding the Gd atom, its intrinsic disorder value was ~5× and ~25× higher than those of the Co and Sb, respectively, denoting that Gd has a strong rattling behavior with an Einstein temperature of $\theta_{E}$ = 67(2) K. As a result, an ultra-low thermal conductivity of 0.89 W/m·K at 773 K was obtained, leading to a thermoelectric efficiency *zT* of 0.5 at 673 K.

## 1. Introduction

In recent times, increasing energy demand has been observed as an unstoppable trend, making it necessary to seek new energy sources or to increase the efficiency of old sources. Approximately two-thirds of energy production is wasted as heat, a frustratingly significant contribution that prevents greater energy efficiency. Thermoelectric devices can transform useless heat into electricity directly and reversibly, and they may reduce the portion of wasted energy [1,2]. Thermoelectric generators are no-moving-parts-type devices; they require less maintenance and are much more reliable than most power-generation systems. However, before this technology becomes widespread, the conversion efficiency of thermoelectric devices needs to be increased above the current ~5% [3,4,5]. This thermoelectric efficiency depends on the material thermoelectric figure of merit, $zT=S^{2}\sigma T/\kappa$, where S stands for Seebeck coefficient, σ for electrical conductivity, κ for total thermal conductivity, and T for absolute temperature. Therefore, there is intense demand for novel materials with higher efficiency (zT) and other relevant properties (stability, thermal expansion, power-factor, price, environmental friendliness, etc.).

Thermoelectric materials are mainly sought among heavily doped narrow semiconductors [6,7]. In particular, the materials with skutterudite structures, derived from CoSb_3_ pnictide, display a promising thermoelectric performance [8,9]. The binary skutterudite CoSb_3_ (or Co_4_Sb_12_) is a narrow-band-gap semiconductor thermoelectric (TE) material offering excellent electrical performance. However, it shows high thermal conductivity due to the high covalency of the Co–Sb chemical bond, resulting in a low zT. Several strategies have been designed to reduce κ in skutterudites; the most extended is the so-called “filling” of the crystal structure [7,10,11,12,13,14,15,16].

The backbone of the skutterudite crystal structure is a framework of corner-sharing (CoSb_6_) octahedra, which is heavily tilted in the three directions of real space. This structure, belonging to the $Im\bar{3}$ space group (no. 204), contains large voids at 2*a* sites, where distinct elements such as rare earth, alkali earth or alkali cations can be lodged in overdimensioned cavities, where the effect of “rattling“ of these filler elements drastically reduces the lattice thermal conductivity in filled skutterudites [8]. In agreement with the PGEC theory (phonon–glass, electron–crystal) [17,18], this “ball in a cage” configuration of the filled skutterudites plays a pivotal role in the basic conditions for high zT values.

In previous works, we synthesized and characterized these compounds under high-pressure conditions, given their metastable character. For example, Co_4_Sb_12_ was stabilized at 3.5 GPa, and we found low thermal conductivities that were attributed to partial Sb deficiency, such as Sb vacancies acting as phonon scatterers [19]. We stabilized La_x_Co_4_Sb_12_, with the La acting as the rattler element [20]. Subsequently, other M_x_Co_4_Sb_12_ pnictide skutterudites were filled with different rare earths (Ce, Yb, Y, mischmetal), alkali earth or alkali elements (Sr, K), which were introduced in the 2*a* skutterudite cages, at hydrostatic pressures of 3.5 GPa at moderate temperatures [21,22,23,24,25].

For a more general picture on the filling process in Co_4_Sb_12_, fundamental knowledge is still missing, such as the atom-radius-size effect for reducing the thermal conductivity. Although large and small M radius sizes were effective in dramatically increasing the acoustic phonon scattering, less information on medium M radius sizes (e.g., M = Gd) is available in the literature. Some exceptional works devoted their attention for Gd-filled skutterudites (Gd_x_Co_4_Sb_12_). In particular, Yang et al. described the thermoelectric performance of high-pressure-obtained Gd_0.12_Co_4_Sb_12_, with the highest zT = 0.52 at 600 K [26], and Liu et al. investigated the thermoelectric properties of Gd_x_Fe_y_Co_4-y_Sb_12_ skutterudites prepared by the melting-annealing method, showing empirically the role of Gd in dramatically reducing the thermal conductivity [27]. However, a more careful structural determination was needed to probe the temperature-dependent lattice dynamics in Gd-filled skutterudites and the rattling effect of Gd atoms for a further comparison with fillers of large and small M radius sizes. Here, we describe a Gd-filled skutterudite (Gd_x_Co_4_Sb_12_) prepared at 3.5 GPa, from which high-angular-resolution synchrotron X-ray diffraction (SXRD) data disclosed conspicuous features that accounted for a substantial decrease in the thermal conductivity in this thermoelectric material.

## 2. Experimental Methods

The composition of Gd_0.5_Co_4_Sb_12_ was prepared by a solid-state reaction under high-pressure and moderate-temperature conditions. A total of 1.2 g of a stoichiometric mixture of the starting elements Gd (99.0%, Alfa Aesar, Kandel, Germany), Co (99 %, ROC/RIC, UK), and Sb (99.5 %, Alfa Aesar, Kandel, Germany) were ground and then conditioned in a niobium capsule (5 mm in diameter), sealed, and inserted in a cylindrical graphite heater. The capsule was manipulated inside an Argon-filled glove box. Reactions were conducted in a piston-cylinder press (Rockland Research Co., West Nyack, NY, USA), at a pressure of 3.5 GPa, at 800 °C for 1 h. Next, the products were quenched down to room temperature and the pressure released. The recovered sample was obtained in the form of hard pieces, which were ground into fine powder for structural characterization or cut with a diamond saw for transport measurements.

High-resolution synchrotron X-ray diffraction (SXRD) patterns were recorded at the beamline ID22 at the European Synchrotron Radiation Facility (ESRF, Grenoble, France). The incident X-ray radiation wavelength was set to 0.35418 Å. The temperature interval between 10 and 1000 K was covered using a He-cooled cryostat and a hot-air blower, with the powder sample loaded into quartz capillaries of 0.5 mm in diameter. The diffraction patterns were collected over the 2θ 1°–40° range with a multi-analyzer stage of 13 Si(111) crystals. High-resolution powder-diffraction patterns were retrieved following the procedure described in [28]. Rietveld refinements were carried out using the *FullProf* program (version 2019), and the peak shape was described using a pseudo-Voigt function [29]. The refinement included the following parameters: scale factors, zero-point error, background coefficients, asymmetry-correction factors, lattice parameters, atomic positions, occupancy factors, and isotropic displacement parameters. Field-effect scanning electron microscopy (FE-SEM) images were obtained in a FEI-Nova microscope (FEI, Eindhoven, Netherlands), with an acceleration potential of 5 kV, coupled to an energy-dispersive X-ray spectrometer (EDX), with an acceleration voltage and acquisition time of 18 kV and 60 s, respectively.

The Seebeck coefficient was obtained using a commercial MMR-technologies system. Measurements were recorded from room temperature up to 800 K, under vacuum. A constantan wire was employed as reference for comparison with bar-shaped skutterudite samples cut with a diamond saw perpendicular to pressing direction. The reproducibility was verified with different contact points and constantan wires. A Linseis LFA 1000 instrument was considered to measure the thermal diffusivity (α) of the samples over a temperature interval 300 ≤ *T* ≤ 800 K by the laser-flash technique. A thin graphite coating was applied to the surface of the pellet to maximize heat absorption and emissivity. The thermal conductivity (κ) was derived using κ = α·C_p_·ρ*_p_*, where C_p_ is the specific heat (410 J/mol·K, see Supplementary Information) and ρ*_p_* is the sample physical density. The sample density was measured considering the Archimedes principle and showed a value of 95(1)% of the theoretical value. The specific heat was estimated using the Dulong–Petit law in the temperature range of 300–800 K (classical limit).

## 3. Results and Discussion

### 3.1. Crystal Structure

**Figure 1:**
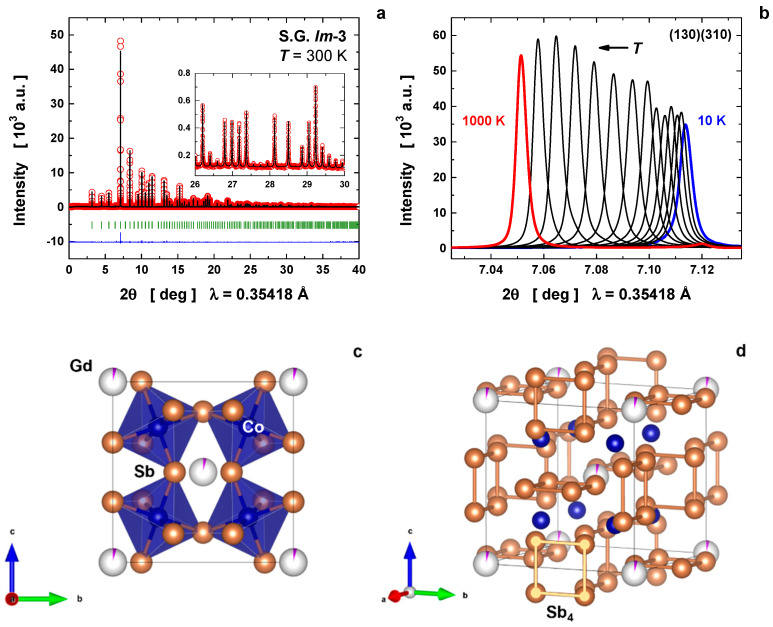
(**a**) Rietveld refinement of the synchrotron X-ray-diffraction pattern of Gd-filled Co_4_Sb_12_ skutterudite at room temperature. (**b**) Temperature evolution in the range 10–1000 K of the main reflection (130)(310). In part (**a**), red open circles are the experimental data, black line denotes the calculated profile, blue line the difference between experimental and calculated data, and dark green bars the Bragg reflections. Crystal-structure representation exhibiting (**c**) the octahedral units (CoSb_6_) and (**d**) the Sb rings (Sb_4_).

The crystalline structure of the as-obtained Gd-filled Co_4_Sb_12_ was probed from high-resolution SXRD data. The unit-cell was indexed at room temperature by following the symmetry restriction of the body-centered space group $Im\bar{3}$ (no. 204, $T_{h}^{5}$) with lattice parameter a = 9.042849(7) Å; a Rietveld plot at RT is shown in Figure 1a. With eight formula per unit-cell (Z = 8), the atoms were distributed as follows: Co at 8*c* ($\bar{3}$) sites (0.25, 0.25, 0.25), Sb at 24*g* (m) sites (0, *y* = 0.33511(3), *z* = 0.15792(3)), and Gd at 2*a* ($m\bar{3}$) sites (0, 0, 0). The crystal structure of the Gd-filled Co_4_Sb_12_ is shown in Figure 1c,d, which highlight the distorted octahedral units (CoSb_6_) and the Sb rings (Sb_4_) formed as a result of the strong octahedral tilting, respectively. It was determined that the nominal composition *x* = 0.5 was not reached; instead a filling fraction of x = 0.033(2) was obtained, leading to the chemical formula Gd_0.033(2)_Co_4_Sb_12_. No secondary (or minor) phases were detected. Table 1 summarizes the refined parameters at room temperature, in addition to the bond lengths Co–Sb, Gd–Sb, and Sb–Sb. The latter had two values for describing the rectangular shape of the ring (Sb_4_).

Next, a precise temperature-dependent evaluation was carried out on the Gd-filled Co_4_Sb_12_ skutterudite to probe its thermal expansion and thermoelastic properties from 10 up to 1000 K. As expected, no phase transition was observed and only the volume expansion was detected, as exemplified in Figure 1b, through shifts to lower angles of the main reflection (130)(310). The raw diffraction data as a function of temperature are also represented in Figure S1 in the Supplementary Information. At 900 and 1000 K, a segregation of some minor, unidentified phase was observed, given the metastable character of this skutterudite. In addition, a Rietveld plot at 800 K is displayed in Figure S2, where the skutterudite is still observed as a single phase.

### 3.2. Thermoelastic Properties

Considering the absence of any structural transition at low and high temperatures in Gd_0.033(2)_Co_4_Sb_12_, we can proceed to evaluate both the thermal expansion and the thermoelastic properties of this compound. The available data cover the temperature interval of 10–1000 K, which enables a second-order expansion of the Grüneisen approximation. In this method, the anharmonic contributions from the lattice vibration are considered and the thermal expansion of the unit-cell volume V(T) has a similar description to the elastic strain and can be second-order expanded, as follows [30,31,32]:

(1) $$V\left(T\right)=V_{0}+\frac{V_{0}\;U\left(T\right)}{Q-b\;U\left(T\right)}$$

such that $V_{0}$ denotes the unit-cell volume at 0 K, $Q=\frac{V_{0}B_{0}}{\gamma^{\prime }}$, $b=\frac{B_{0}^{\prime }-1}{2}$, $B_{0}$ and $B_{0}^{\prime }$ are the bulk modulus and its first derivative in pressure at 0 K, and $\gamma^{\prime }$ describes the Grüneisen parameter. The value $\gamma^{\prime }$ is zero for harmonic crystals, and $\gamma^{\prime }$ ≠ 0 when anharmonic effects occur, typically at high temperatures [33]. The function U(T) represents the internal energy, as derived from the Debye theory [32,34], i.e.,

(2) $$U\left(T\right)=9N_{a}k_{B}T{\left(\frac{T}{\theta_{D}}\right)}^{3}\overset{\theta_{D}/T}{\underset{0}{\int }}\frac{z^{3}}{e^{z}-1}dz$$

where $N_{a}$ means the total number of atoms within the unit-cell (in the case of CoSb_3_, $N_{a}$ = 32), $\theta_{D}$ denotes the Debye temperature, and $k_{B}$ and T maintain their usual meaning in physics.

**Figure 2:**
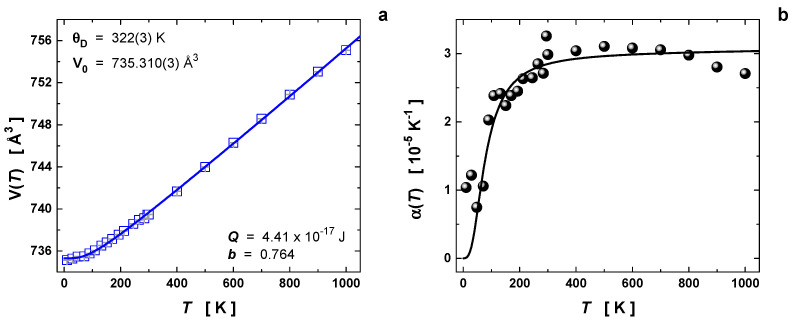
(**a**) Thermal expansion of the unit-cell volume V(T), such that the solid line is the second-order Grüneisen approximation to the zero-pressure equation of state. (**b**) Thermal-expansion coefficient α(T), where the spheres were obtained by numerical first derivative of the unit-cell volume and the black solid line was calculated from the fitted-unit-cell volume expansion using Equation (1).

Using the previous equations, we fitted the volume expansion with the temperature in Figure 2a, where the following parameters were obtained: $\theta_{D}$ = 322(3) K, $V_{0}$ = 735.310(3) Å^3^, Q = 4.41 × 10^−17^ J, and b = 0.764. The comparison between the experimental and fitted curves showed a good agreement over the entire temperature interval. In this model, Q and b are assumed to be temperature-independent. By taking the literature value for the bulk modulus ($B_{0}$) in CoSb_3_ of 100(4) GPa [25], the Grüneisen parameter ($\gamma^{\prime }$) can be estimated around 1.67(5), which partially agrees with the value 1.11(1) for CoSb_3_ extracted from the compressibility data at a constant temperature [35]. Nevertheless, a larger value indicates anharmonic bonding and stronger phonon–phonon interactions, which account for the reduced lattice thermal conductivity [36]. In addition, the first derivative $B_{0}^{\prime }$ is evaluated as 2.5(3). This result demonstrates that the anharmonicity of the lattice vibrations at high temperatures may be an important parameter to describe the thermoelectric performance in filled skutterudites.

From the first derivative of the unit-cell volume, the thermal-expansion coefficient α(T) can be evaluated [31,34], as follows:

(3) $$\alpha \left(T\right)=\frac{1}{V}\frac{dV}{dT}$$

Figure 2b compares the experimental values for α(T) (numerical first derivative of the experimental points) and the calculated thermal-expansion coefficient from the fitting using Equation (1). One may observe that, for temperatures above 800 K, the experimental points started to depart from the calculated thermal-expansion coefficient, possibly due to the internal instabilities of the filler element Gd, which can result in thermal decomposition for temperatures higher than 800 K [19,22], as was also observed from the SXRD data (Figure S1).

### 3.3. Mean-Square Displacement

The Debye approximation can be also used to describe the thermal evolution of the principal thermal-vibration parameters after conversion to the mean-square displacements (MSDs or $U_{eq}$, in units of Å^2^) [37,38,39], as defined by:

(4) $$U_{eq}\left(T\right)=d_{s}^{2}+\frac{3\hbar^{2}T}{mk_{B}\theta_{D}^{2}}\left[\;\frac{T}{\theta_{D}}\overset{\theta_{D}/T}{\underset{0}{\int }}\frac{z}{e^{z}-1}dz+\frac{\theta_{D}}{4T}\;\right]$$

where $d_{s}^{2}$ is the intrinsic disorder (also known as the static displacement at 0 K), m the atom’s mass, and ℏ the reduced Planck constant. This Debye-model evaluation is more appropriate for Co and Sb atoms because such atoms constitute the framework (or the lattice) of the skutterudite structure (see Figure 1c).

The Co atom exhibited an intrinsic disorder of 1.65(7) × 10^−3^ Å^2^ and a Debye temperature of $\theta_{D}$ = 393(1) K. On the other hand, the Sb atom showed an intrinsic disorder of 5.72(8) × 10^−4^ Å^2^ and a Debye temperature of $\theta_{D}$ = 240(1) K. These values for $\theta_{D}$, averaged by the atomic masses of cobalt and antimony, provide a Debye temperature for the framework of $\theta_{D}$ = 265(2) K, which agrees with previous values for CoSb_3-δ_ ($\theta_{D}$ = 262 K) [19] and other partially filled skutterudites ($\theta_{D}$ = 265–274 K) [40], which were also extracted from MSD analyses. Our result of $\theta_{D}$ = 265(2) K is in partial agreement with the Debye temperatures estimated from the heat-capacity curves ($\theta_{D}$ = 280–294 K) [40]. However, the Debye temperature evaluated from both the volume expansion and the MSDs displayed quite different values, i.e., 322(3) and 265(2) K, respectively. This difference originates in the evaluation method of the Debye temperature and the available temperature range; it was also noticed by Vočadlo et al. for ε-FeSi [31]. The thermal-expansion fitting of the unit-cell volume is indeed very sensitive to the temperature range and anharmonic effects [31], which may explain such differences and suggests that the application of only harmonic Debye approximation may underestimate the $\theta_{D}$ value in skutterudites.

**Figure 3:**
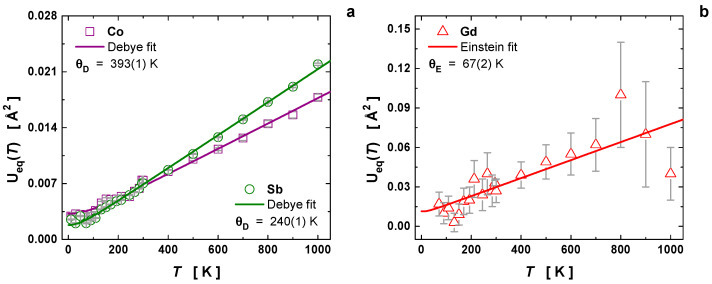
Temperature dependence of the mean-square displacements $U_{eq}\left(T\right)$ (or MSDs in units of Å^2^) for the atoms (**a**) Co, Sb and (**b**) Gd. The Co–Sb framework was evaluated using the Debye approximation, while the dynamic behavior of the filler Gd was fitted to the Einstein model.

The Gd atom exhibits weaker chemical bonds at 2*a* sites (voids), enabling its rattling behavior [41] for the benefit of acoustic phonon scattering, thus reducing the thermal conductivity [23,40,42]. In such cases, the normal vibrations can be treated as independent oscillators according to the Einstein approximation, as follows [24,43]:

(5) $$U_{eq}\left(T\right)=d_{s}^{2}+\frac{\hbar^{2}}{2mk_{B}\theta_{E}}\coth\left(\frac{\theta_{E}}{2T}\right)$$

where $\theta_{E}$ denotes the Einstein temperature. The fitted value for the intrinsic disorder $d_{s}^{2}$ approaches 9.1(2) × 10^−3^ Å^2^, which is about ~5× and ~25× higher than the values for the Co and Sb, respectively. This result shows that Gd has a strong rattling effect at the 2*a* voids, and an Einstein temperature of $\theta_{E}$ = 67(2) K. This low value agrees with those for typical fillers of rare-earth elements, normally ranging from 54–88 K [20,22,40]. Specially, the $\theta_{E}$ value for Gd is close to that for Eu, i.e., $\theta_{E}$ = 68(2) K [40], both elements having similar atomic masses and medium M radius size. In Figure 3b, a slope change in the Gd MSD is shown at 800 K, which may be associated with internal instabilities of the filler in view of the high $U_{eq}$ value of 0.1(6) Å^2^, or the onset of skutterudite decomposition.

### 3.4. Local Atomic Bonding

**Figure 4:**
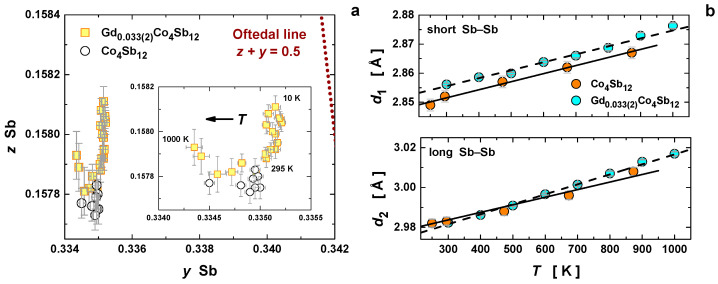
(**a**) Oftedal plot with the Oftedal line (red dotted line, such that z+y = 0.5) comparing Gd-filled and unfilled Co_4_Sb_12_. (**b**) Temperature dependence of the short and long Sb–Sb distances in the (Sb_4_) ring for both Gd-filled and unfilled Co_4_Sb_12_.

As established in the literature [24,44,45], the skutterudite-type structure belonging to the space-group $Im\bar{3}$ is fully described by three parameters, namely the lattice constant a and the Sb fractional coordinates y and z. While the lattice constant is sensitive to the filling fraction, the Sb fractional coordinates can be used to probe the topology of the (Sb_4_) ring [23]. For a square ring, where the short (*d*_1_) and long (*d*_2_) Sb–Sb distances have the same length, the coordinates are constrained to z+y = 0.5 (Oftedal line) [46]. Figure 4a displays the Oftedal plot (z vs. y) for both Gd-filled and unfilled Co_4_Sb_12_ (from [19]). Two tendencies can be observed: (1) the incorporation of the filler brings the Sb ring closer to the Oftedal line; and (2) the temperature increase induces a ring deformation from a square to a more rectangular shape. This behavior was also seen by Hanus et al. for Yb-filled Co_4_Sb_12_ [45].

The different temperature evolutions of the (Sb_4_) ring-bond distances (Figure 4b) were analyzed using the local-linear-expansion coefficients (Table 2), calculated as $\alpha_{l}\left(T\right)=\frac{1}{l}\frac{dl}{dT}$. These provide an indication of the electron density between the atoms and the respective bond strength, as weaker bonds show a more pronounced expansion with temperature [45]. Here, a significant increase in the short Sb–Sb bond distance at room temperature was observed after filling; however, the local expansion coefficient remained analogous to unfilled Co_4_Sb_12_ prepared under high pressure. The long Sb–Sb bonds displayed a similar distance in the unfilled and filled samples, while the local thermal-expansion coefficient in the Gd-filled Co_4_Sb_12_ was much larger, suggesting a much weaker covalent interaction. This was in contrast with previously reported local thermal expansion coefficients of Yb_0.3_Co_4_Sb_12_, in which the significant weakening of short Sb–Sb distances led to a more squared bond order of short and long Sb–Sb interactions. This lengthening is related to the change in the electronic structure and population of antibonding states, leading to band convergence, the core of the excellent thermoelectric performance of filled Co_4_Sb_12_ [47]. By contrast, while Gd-filled Co_4_Sb_12_ still shows an increase of short Sb–Sb bond distances, the structure still retains a much stronger short Sb–Sb bond than its long Sb–Sb bond, which is weakened. This is related to the low filling fraction and different electronic interactions of Gd^3+^ and Yb^3+/2+^ within the skutterudite structure, elucidating different mechanisms for filled skutterudites with small and medium M radius sizes. Furthermore, as reported previously, Co–Sb bond nature remains unaltered after filling.

### 3.5. Microstructure

**Figure 5:**
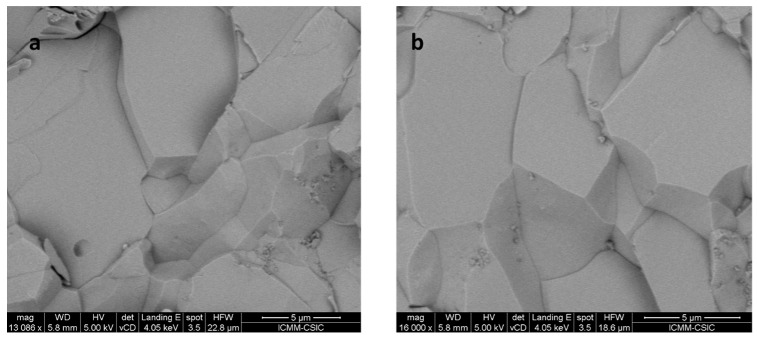
SEM image of Gd_0.033(2)_Co_4_Sb_12_ exhibiting well-sintered grains with no porosity due to the high-pressure preparation, with magnification (**a**) 13,086× and (**b**) 16,000×.

The texture of the as-grown Gd_x_Co_4_Sb_12_ pellets can be observed in the FE–SEM images shown in Figure 5. The material has compact microcrystals, each of which are presumably single-crystalline and sintered to the neighboring crystals for accounting for the compactness of this high-pressure-sintered sample, with a physical density of 95(1)% of the theoretical value. The microstructure clearly shows the grain boundaries, which play a role to the carrier and phonon transport. The high-pressure and high-temperature procedure favor the grains’ growth process significantly. The analysis carried out from EDX is included in the Supplementary Information, as Figure S3.

### 3.6. Thermoelectric Performance

**Figure 6:**
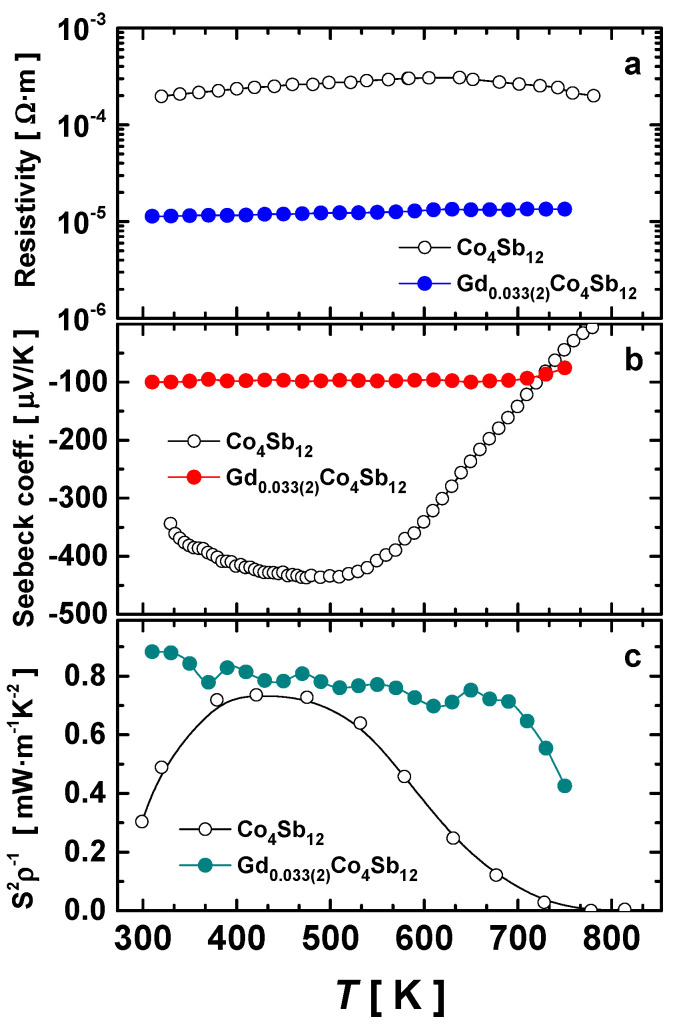
Temperature dependence of the: (**a**) resistivity, (**b**) Seebeck coefficient, and (**c**) power factor (S^2^ρ^−1^) of the Gd-filled skutterudite obtained under high-pressure conditions at 3.5 GPa.

The three main thermoelectric transport properties of the unfilled Co_4_Sb_12_ and the Gd_0.033(2)_Co_4_Sb_12_ composition are displayed in Figure 6. There was a significant reduction in the resistivity (Figure 6a) with the addition of the filler element, even in these small quantities, dropping down to ~10^−5^ Ω.m throughout the entire measurement range and showing a steady behavior, like that of other filled skutterudites [23]. Moreover, these values are slightly better than those reported for both Gd_0.09_Co_4_Sb_12_ and Gd_0.12_Co_4_Sb_12_ prepared at 4 GPa, which are between 2 × 10^−5^ Ω·m and 4 × 10^−5^ Ω·m [26]. This resistivity is also lower than that reported for Gd-filled CoSb_3_ prepared by a non-equilibrium melt-spinning process, which is around 2 × 10^−5^ Ω·m [48]. The Seebeck coefficient showed a significant reduction at room temperature compared to the −350 µV/K of the unfilled Co_4_Sb_12_, which reduced to −100 µV/K for the Gd-filled compound (Figure 6b). This drastic change in the Seebeck coefficient cannot only be attributed to the charge-transfer process from the filler element to the skutterudite framework because the refined filling fraction is only x = 0.033(2). Instead, the change in this transport parameter is affected by both the charge transfer and the change along the band structure induced by the filler element, the Gd atom. The lower Seebeck coefficient shown by this composition compared to that reported for the Gd_x_Co_4_Sb_12_ specimen prepared at 4 GPa [26] (−260 µV/K at room temperature), together with the reduction in the resistivity, suggest that the charge transfer is more prominent in this sample. Nevertheless, this is inconsistent with the lower filling fraction values and is discussed below. The calculated power factor can be seen in Figure 6c, exhibiting higher values than that of the undoped compound, but lower than those reported for other Gd-filled compounds [26].

**Figure 7:**
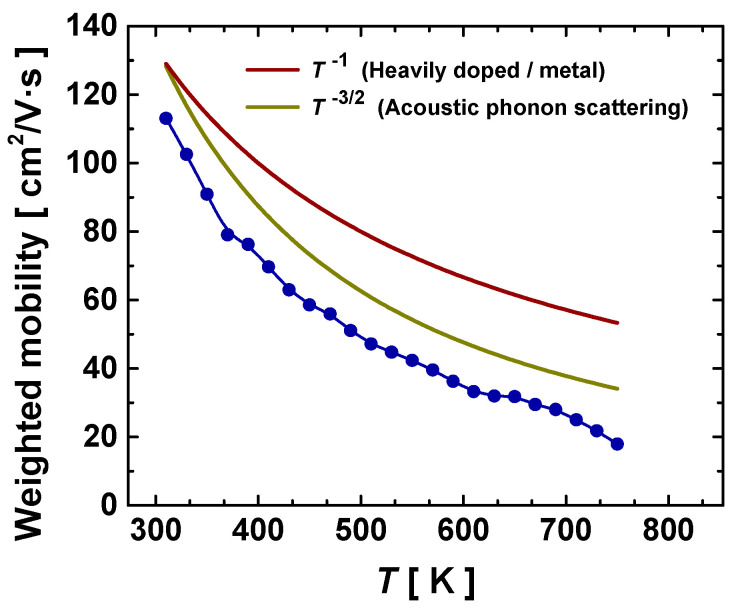
Calculated weighted mobility of the Gd-filled skutterudite obtained under high-pressure conditions at 3.5 GPa (dark-blue symbols). The brown and dark-yellow lines exhibit trends associated with different behaviors, heavily doped and acoustic-phonon scattering, respectively.

The weighted mobility [49] of the Gd-filled compound, as estimated from the experimental Seebeck coefficient and resistivity, is shown in Figure 7. This mobility gives similar information on the carrier mobility to the Hall mobility; therefore, it is possible to extract some useful hints about the behavior of the charge carriers from the analysis of this plot. The main insight is that the electronic transport in this compound is limited by acoustic phonon scattering, an effect that can be noted by the decrease in the weighted mobility with temperature following a $T^{-3/2}$ relation. The weighted mobility of this Gd-filled derivative is higher than that observed for other filled skutterudites prepared by the same method [50]. Taking this into account, our samples may have presented a lower carrier concentration than expected, while the Seebeck coefficient was not as enhanced by the band convergence as in another higher-filling-fraction Gd Co_4_Sb_12_. This agrees with the relationship among the filling fraction, doping level of the antibonding states, and the conduction-band convergence, which foresees a low filling fraction value as that extracted from the SXRD. Indeed, the structural analysis points towards the same argument, as the short Sb–Sb bonds still displayed lower values of thermal expansion and a much stronger interaction than the long Sb–Sb bonds, while the opposite behavior would encourage band convergence [45].

**Figure 8:**
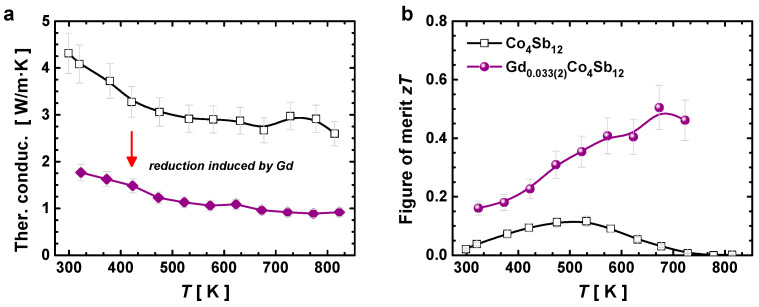
(**a**) Total thermal conductivity and (**b**) thermoelectric figure of merit (*zT*) of the unfilled and the Gd-filled compound obtained under high pressure at 3.5 GPa.

The total thermal conductivity is displayed in Figure 8a. There was a major reduction in the thermal conductivity of the Gd-filled composition compared to the unfilled composition, as reported previously for other filler elements [20,21,22]. The thermal conductivity was as low as 0.89 W/m.K at 773 K, which was lower than the minimum of ~3 W/m.K reported for other Gd-filled skutterudites synthesized at high pressure [26], and the ~1.5–2 W/m.K reported for other doped skutterudites [51,52]. This decrease in the thermal conductivity was a consequence of the rattling behavior of the Gd atom, which, as we described previously, showed an intrinsic disorder parameter ~5× and ~25× higher than those of the Co and Sb atoms, respectively. Lastly, the calculated thermoelectric figure of merit (*zT*) is shown in Figure 8b. A maximum value (*zT_max_*) of 0.5 was obtained at 673 K, which was approximately equal to that reported for both Gd_0.09_Co_4_Sb_12_ and Gd_0.12_Co_4_Sb_12_ compounds [26], but shifted towards higher temperatures.

## 4. Conclusions

In short, the Gd-filled skutterudite, Gd_x_Co_4_Sb_12_, was successfully synthesized in the one-step method under pressure in a piston-cylinder press at 3.5 GPa and at a moderate temperature of 800 °C. Structural characterizations were performed using the SXRD, from which a filling fraction of x = 0.033(2) and an average <Gd–Sb> bond length of 3.3499(3) Å were estimated. The lattice thermal expansion and lattice anharmonicity were studied via temperature-dependent SXRD. A Debye temperature of 322(3) K, from the fitting of the unit-cell volume expansion using the second-order Grüneisen, was derived with a Grüneisen parameter $\gamma^{\prime }$ = 1.67(5). The Debye temperature was additionally evaluated from the mean-square displacements of the Co and Sb, leading to a value of 265(2) K. This difference means that the application of the harmonic Debye theory underestimates the Debye temperature in skutterudites. At the Gd sites, its intrinsic disorder value was ~5× and ~25× higher than those of the Co and Sb, respectively, suggesting that Gd has strong rattling with an Einstein temperature of $\theta_{E}$ = 67(2) K. This rattling behavior encourages an ultra-low thermal conductivity, of 0.89 W/m·K at 773 K, as inferred from the acoustic-phonon scattering, which consequently led to a thermoelectric efficiency (*zT_max_*) of 0.5 at 673 K.

## Figures and Tables

**Table 1:** Refined structural parameters of the Gd-filled Co_4_Sb_12_ skutterudite from SXRD data at room temperature. *Abbreviations*: *U*_eq_ is the equivalent mean-square displacement, Site Occ. the site occupancy, and $\rho_{t}$ the theoretical density.

Atom	Wyckoff	*x*	*y*	*z*	*U*_eq_ (10^−3^ Å^2^)	Site Occ.
Co	8*c*	0.25	0.25	0.25	7.4(1)	1
Sb	24*g*	0	0.33511(3)	0.15792(3)	7.05(4)	1
Gd	2*a*	0	0	0	28(7)	0.033(2)
Unit-cell parameters		Bond lengths			Reliability factors	
a (Å)	9.042849(7)	*d*_0_ (Co–Sb) (Å)	2.5291(3)		*R*_p_ (%)	6.30
V (Å^3^)	739.4619(10)	*d*_1_ (Sb–Sb) (Å)	2.8561(4)		*R*_exp_ (%)	4.94
$\rho_{t}$ (g·cm^−3^)	7.644(1)	*d*_2_ (Sb–Sb) (Å)	2.9823(4)		*R*_wp_ (%)	6.86
		*d*_3_ (Gd–Sb) (Å)	3.3499(3)		*R*_Bragg_ (%)	1.92

**Table 2:** Linear thermal-expansion coefficients ($\alpha_{l}$ ) evaluated for the bonds Co–Sb, Sb–Sb, and M–Sb in both unfilled [19] and filled (M = Gd and Yb [45]) Co_4_Sb_12_ skutterudites, normalized with respect to the 300-kelvin bond length.

Structural Parameter	$\alpha_{l}\;(\times 10^{-6}\;K^{-1})$		
	Co_4_Sb_12_	Gd_0.033(2)_Co_4_Sb_12_	Yb_0.3_Co_4_Sb_12_
*d*_0_ (Co–Sb)	8.9	9.1	9.1
*d*_1_ (Sb–Sb)	9.7	9.6	12.6
*d*_2_ (Sb–Sb)	12.7	16.8	15.9
*d*_3_ (M–Sb)	-	7.3	8.6

## Data Availability

The datasets used and/or analyzed are available from the corresponding author on reasonable request.

## Supplementary Materials

The following supporting information can be downloaded at: https://www.mdpi.com/article/10.3390/ma16010370/s1, Figure S1. (a) Temperature-dependent SXRD patterns of Gd_x_Co_4_Sb_12_ skutterudite recorded in the temperature range 10−1000 K. An unidentified minor phase is segregated at 900 and 1000 K, as observed in the 7−8° angular region. Selected 2θ ranges are shown, namely: 2θ 12.9°−14.1° (b) and 2θ 18.3°−19.5° (c) to elucidate the lattice thermal expansion. Figure S2. Rietveld plot from synchrotron X-ray diffraction data of Gd-filled Co_4_Sb_12_ skutterudite at 800 K. Red crosses are the experimental data, the black line denotes the calculated profile, the blue line is the difference between experimental and calculated data, and dark green bars the Bragg reflections. The inset shows the quality of the fit in the high angular region between 26° and 30°. Figure S3. EDX analysis coupled to the FE-SEM images. A typical EDX spectrum, showing the region where it has been collected. The atomic composition is close to 0.033:4:12 for the Gd:Co:Sb ratio. Figure S4. Specific heat of Gd_0.033(2)_Co_4_Sb_12_ composition in the temperature range 5–310 K.
