# Supplementary material for: Thermal Expansion and Rattling Behavior of Gd-Filled Co4Sb12 Skutterudite Determined by High-Resolution Synchrotron X-ray Diffraction
Source: Materials (Basel). 2022 Dec 30;16(1):370. doi: 10.3390/ma16010370 (PMC9822039; doi:10.3390/ma16010370)
Supplement: Supplementary file 1 [file materials-16-00370-s001.zip › materials-2070402-supplementary.pdf]

SUPPLEMENTARY INFORMATION

Thermal expansion and rattling behavior of Gd-filled Co_4Sb_{12} skutterudite determined by high-resolution synchrotron X-ray diffraction

João E. F. S. Rodrigues [1,2,*], Javier Gainza [1], Federico Serrano-Sánchez [1], Romualdo S. Silva, Jr. [3], Catherine Dejoie [2], Norbert M. Nemes [4], Oscar J. Dura [5], José L. Martínez [1] and José Antonio Alonso [1,*]

[1] Instituto de Ciencia de Materiales de Madrid (ICMM), Consejo Superior de Investigaciones Científicas, Sor Juana Inés de la Cruz 3, E-28049 Madrid, Spain.
[2] European Synchrotron Radiation Facility (ESRF), 71 Avenue des Martyrs, 38000 Grenoble, France.
[3] Department of Physics, Federal University of Sergipe, 49100-000, São Cristóvão-SE, Brazil.
[4] Departamento de Física de Materiales, Universidad Complutense de Madrid, E-28040 Madrid, Spain.
[5] Departamento de Física Aplicada, Universidad de Castilla-La Mancha, Ciudad Real, E-13071, Spain.

***** Correspondence: rodrigues.joaoelias@gmail.com (J.E.F.S.R.); ja.alonso@icmm.csic.es (J.A.A.).

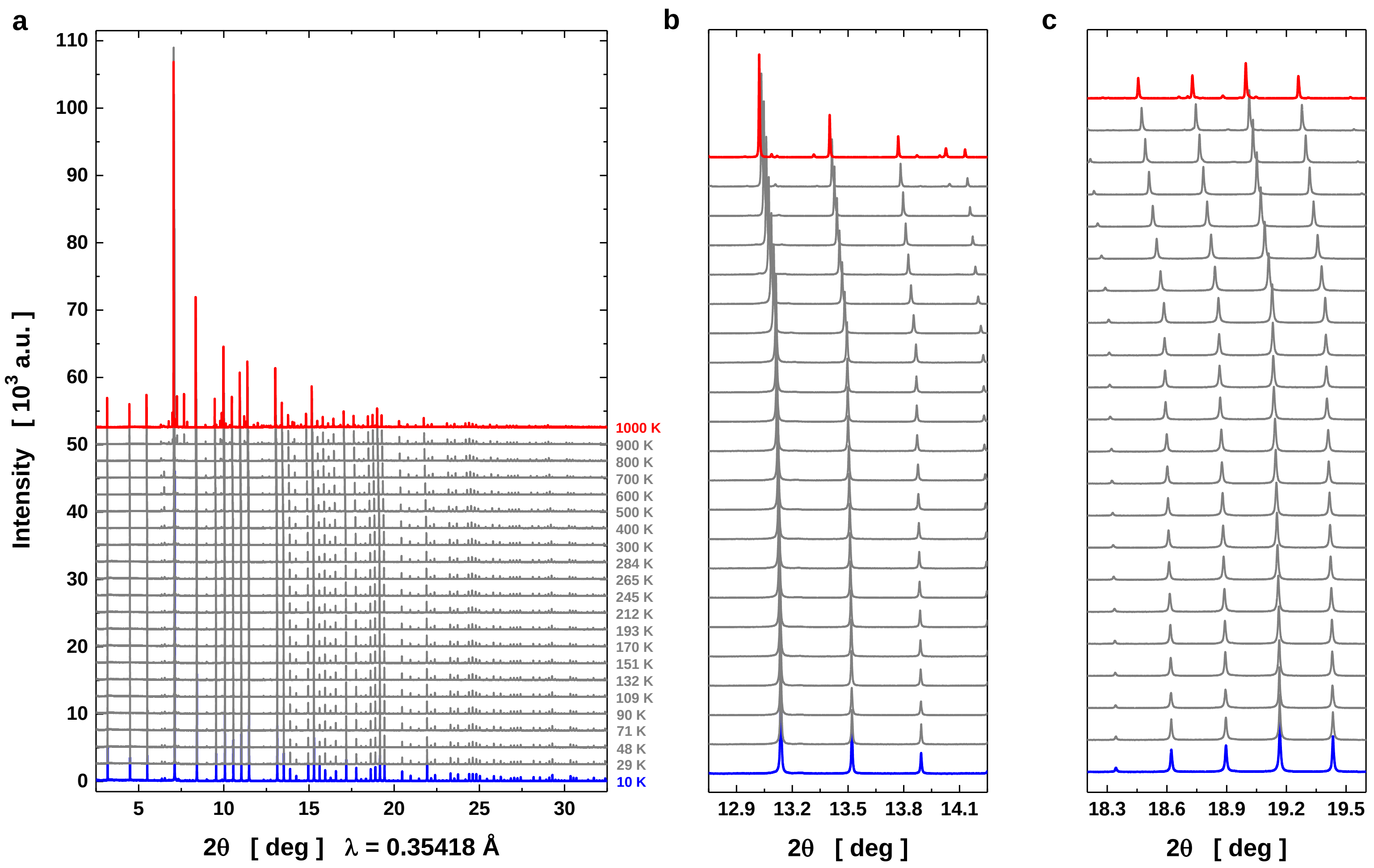

Figure S1. (a) Temperature-dependent SXRD patterns of $Gd_xCo_4Sb_{12}$ skutterudite recorded in the temperature range 10−1000 K. An unidentified minor phase is segregated at 900 and 1000 K, as observed in the 7-8° angular region. Selected 2θ ranges are shown, namely: 2θ 12.9°−14.1° (b) and 2θ 18.3°−19.5° (c) to elucidate the lattice thermal expansion.

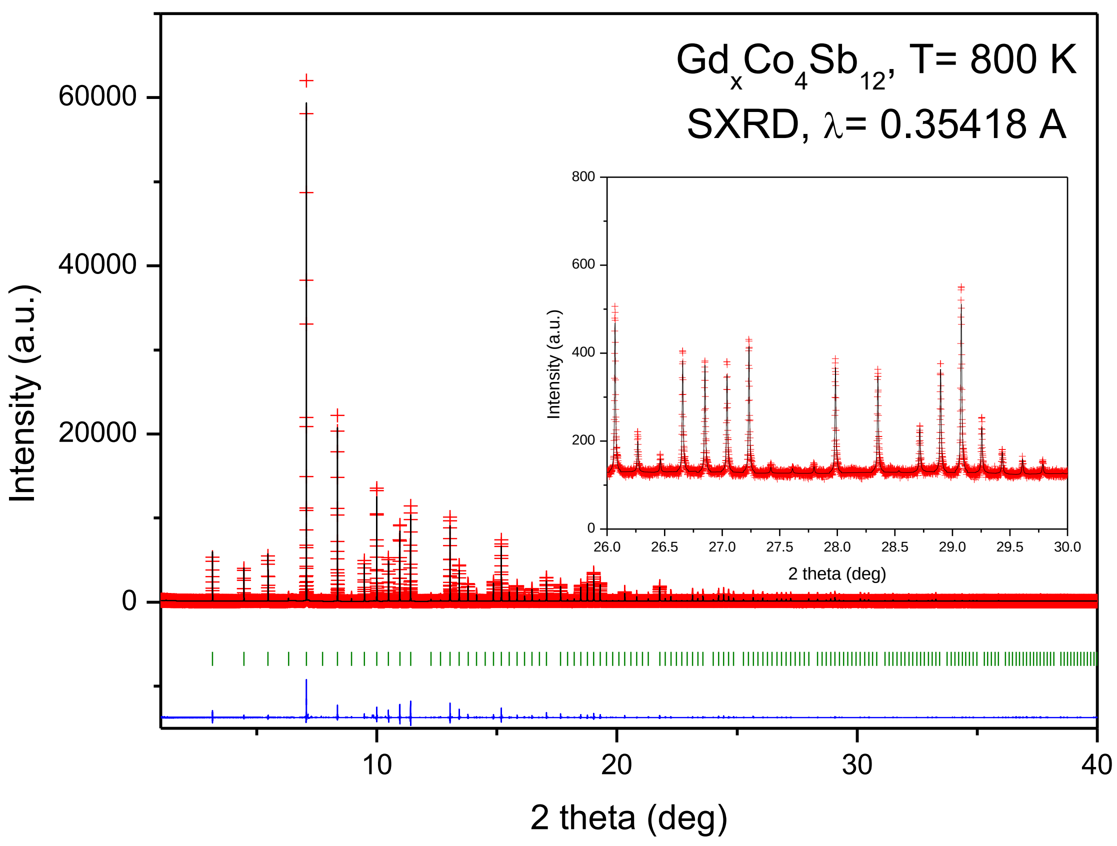

Figure S2. Rietveld plot from synchrotron X-ray diffraction data of Gd-filled Co_4Sb_{12} skutterudite at 800 K. Red crosses are the experimental data, the black line denotes the calculated profile, the blue line is the difference between experimental and calculated data, and dark green bars the Bragg reflections. The inset shows the quality of the fit in the high angular region between 26º and 30º.

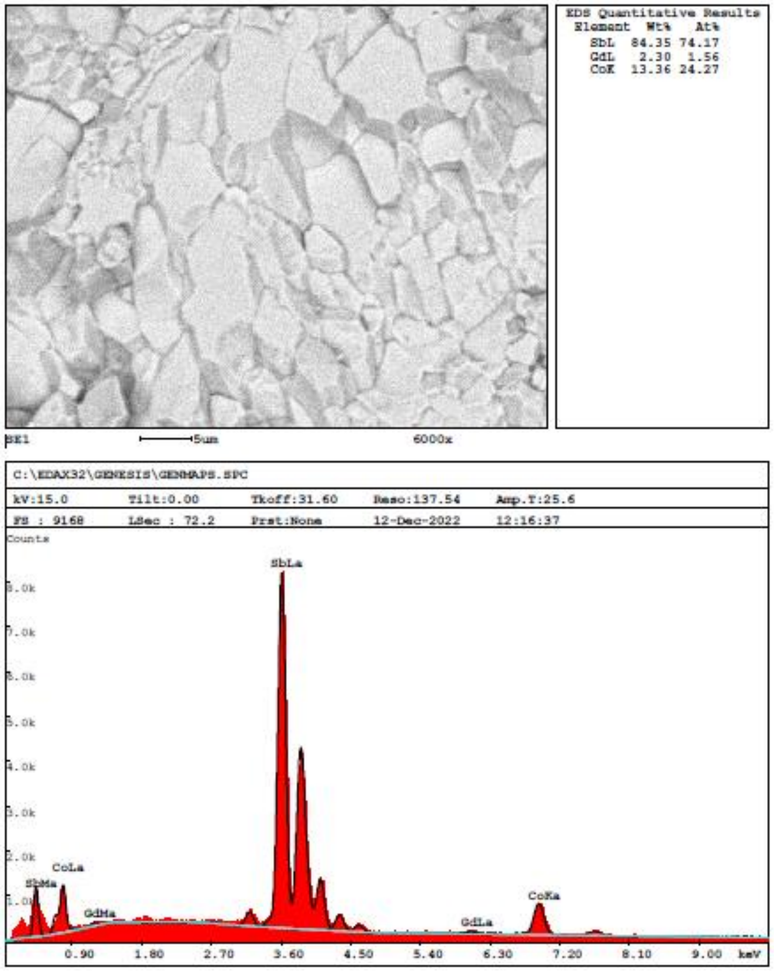

Figure S3. EDX analysis coupled to the FE-SEM images. A typical EDX spectrum, showing the region where it has been collected. The atomic composition is close to 0.033:4:12 for the Gd:Co:Sb ratio.

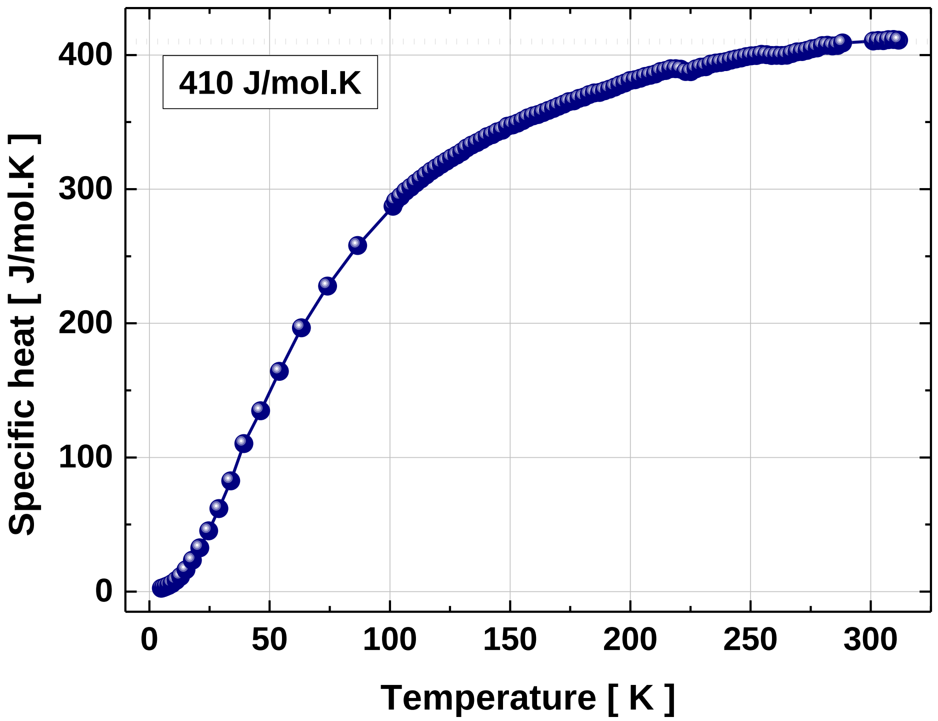

Figure S4. Specific heat of $Gd_{0.033(2)}Co_4Sb_{12}$ composition in the temperature range 5–310 K.
